# Nickel Oxy-Hydroxy/Multi-Wall Carbon Nanotubes Film Coupled with a 3D-Printed Device as a Nonenzymatic Glucose Sensor

**DOI:** 10.3390/bios13060646

**Published:** 2023-06-13

**Authors:** Murillo N. T. Silva, Raquel G. Rocha, Eduardo M. Richter, Rodrigo A. A. Munoz, Edson Nossol

**Affiliations:** Institute of Chemistry, Federal University of Uberlândia, Uberlândia 38400-902, MG, Brazil; murilloneia@hotmail.com (M.N.T.S.); raquel_rocha1793@hotmail.com (R.G.R.);

**Keywords:** nickel oxy-hydroxy, carbon nanotube, glucose, electrochemical sensor, batch injection analysis

## Abstract

A rapid and simple method for the amperometric determination of glucose using a nanocomposite film of nickel oxyhydroxide and multi-walled carbon nanotube (MWCNTs) was evaluated. The NiHCF)/MWCNT electrode film was fabricated using the liquid–liquid interface method, and it was used as a precursor for the electrochemical synthesis of nickel oxy-hydroxy (Ni(OH)_2_/NiOOH/MWCNT). The interaction between nickel oxy-hydroxy and the MWCNTs provided a film that is stable over the electrode surface, with high surface area and excellent conductivity. The nanocomposite presented an excellent electrocatalytic activity for the oxidation of glucose in an alkaline medium. The sensitivity of the sensor was found to be 0.0561 μA μmol L^−1^, and a linear range from 0.1 to 150 μmol L^−1^ was obtained, with a good limit of detection (0.030 μmol L^−1^). The electrode exhibits a fast response (150 injections h^−1^) and a sensitive catalytic performance, which may be due to the high conductivity of MWCNT and the increased active surface area of the electrode. Additionally, a minimal difference in the slopes for ascending (0.0561 µA µmol L^−1^) and descending (0.0531 µA µmol L^−1^) was observed. Moreover, the sensor was applied to the detection of glucose in artificial plasma blood samples, achieving values of 89 to 98% of recovery.

## 1. Introduction

Fast and accurate quantification of glucose is vital in many areas, such as the food industry, biotechnology, public health, and clinical diagnostics [1,2]. Several glucose and similar biomolecules-sensing devices and methodologies have been developed in recent decades, which can be generally divided into optical techniques and electrochemical techniques [3]. An optical detection system including fluorescence (FL), colorimetry (CL), localized surface plasmon resonance (LSPR), and surface-enhanced Raman scattering (SERS) had excellent success when implemented without interfering agents, yielding high selectivity [4] for the detection of several biomolecules, including creatinine [5], cardiac troponin I [6], alanine aminotransferase [7], acetylcholine [8], cholesterol [9,10], aflatoxin B1 [11], collagen [12], and p-cresol [13]. In 2021, Lee et al. [6] used multi-functional DNA (MF-DNA) compounds in Au nanocrystal as a dual mode cardiac troponin 1 biosensor using an electrochemical method (EC) and a localized surface plasmon resonance (LSPR) method. Al-Rubaye (2018) developed a highly sensitive analytical method of total internal reflection ellipsometry (TIRE) combined with the LSPR phenomenon in nano-structured gold films for the detection of aflatoxin B1 and M1 in direct assay with specific aptamers immobilized on the surface of gold [11]. Recently, Ujah et al. (2023) successfully demonstrated the detection of glucose using a simple and fast method was used to fabricate highly sensitive refractive index sensors based on localized surface plasmon resonance (LSPR) [14]. Those biosensors exhibit different pros and cons concerning sample preparation, operation time, labeling, sensitivity, cost, and portability. The electrochemical fabrication of glucose sensors has more advantages such as low cost, easy fabrication, high sensitivity, and continuous monitoring [15].

Previous studies have shown the utilization of electrochemical enzymatic glucose biosensors based on the use of glucose oxidase (GO_X_) or glucose dehydrogenase (GD_H_) enzymes. However, due to the intrinsic properties of enzymes, the biosensor can be easily affected by pH, temperature, and/or humidity. Moreover, the immobilization of enzymes on electrode surfaces requires laborious steps [16]. Additionally, a glucose sensor based on enzymes is easily exposed to harsh thermal and chemical conditions during fabrication and storage, and the enzyme synthesis and immobilization onto the electrode surface are often complex and unstable for long-term or repeatable usage [17,18]. Considering the intrinsic drawbacks of the glucose enzyme-based biosensor, non-enzymatic glucose sensors have been studied as a promising alternative for the electrochemical determination of glucose. These approaches offer some advantages, such as better stability and reproducibility, fast response, and low cost in the construction of miniaturized devices [19,20].

Previous studies are reported in the literature using non-enzymatic sensors for the determination of glucose. In particular, fueled by the fast advances in nanotechnology, an increasing number of researchers have been exploring the production of chemically-modified electrode alternative materials, such as novel nanoscale metal alloys [21], bimetallic MOFs [22], transition metals [23], metal oxides [24], hydroxides [1], and spinel metal cobaltite materials [25]. The combination of conductive materials with metal compounds, such as carbon nanomaterials with metal hydroxides, has promoted synergetic properties showing an increase in analytical performance due to the enhancement of the conductivity of electrode materials [24,26].

Carbon nanotubes (CNTs) have been explored for their application in electrocatalysis and the construction of chemical sensors/biosensors because of their high electrical conductivity, chemical stability, and remarkable electronic/mechanical properties [26,27]. Additionally, CNTs have been reported to be synthesized from waste resources [28,29] and have successfully demonstrated applications in the electrochemical detection of various analytes [30]. Additionally, Ni-based materials have been widely utilized as a functional component with which to prepare highly effective electrocatalysts for glucose, due to the intense catalytic action of the NiII/NiIII redox process for the transformation of glucose to glucolactone. These properties depend on the formation of Ni(OH)_2_/NiOOH in an alkaline medium. Nickel oxy-hydroxy-modified electrodes have high electrocatalytic efficiency toward the oxidation of glucose via the cyclic mediation electron-transfer process in alkaline solutions [31]. In comparison with other materials, nickel is naturally abundant, has low toxicity, presents high stability in an alkaline medium, and is an alternative to high-cost noble metals [32]. Regardless of the initially synthesized form, in an alkaline medium, the surface of NiO is easily converted into an Ni(OH)_2_/NiOOH redox couple during electrocatalysis. For example, a nickel hexacyanoferrate (NiHCF) modified electrode, when cycled between 0.0 and 0.9 V in NaOH, can be converted into Ni(OH)_2_/NiOOH [33,34]. The widely used NiO is also activated by OH^-^ in the solution in the form of Ni(OH)_2_ [32].

A promising strategy for the formation of nanocomposites is the use of the biphasic liquid–liquid method. This system provides a simple procedure with which to assemble previously synthesized materials or with which they can be both synthesized into organized arrays or thin films that can be easily deposited over any kind of ordinary conductive substrate for the production of modified electrodes [35,36].

In this work, we present a novel strategy to synthesize a thin film nanocomposite between nickel oxyhydroxide and a multi-walled carbon nanotube (MWCNT) for use as a non-enzymatic glucose sensor. Firstly, an NiHCF/multi-walled carbon nanotube (MWCNT) was synthesized through the biphasic liquid–liquid system, followed by the electrochemical conversion into nickel oxyhydroxide/MWCNT in an alkaline medium. The nanocomposite was characterized by Raman spectroscopy, scanning electron microscopy (SEM), X-ray energy dispersed spectra (EDS), cyclic voltammetry, and electrochemical impedance spectroscopy. The electrochemical performance of the modified electrode for the detection of glucose was investigated and the sensing features, such as low detection limit and good selectivity, can be highlighted.

## 2. Materials and Methods

### 2.1. Chemical and Samples

All solutions were prepared using deionized water obtained from a Milli-Q-MilliRho purification system (Millipore, Bedford, MA, USA). Analytical-grade chemicals were employed and used without additional purification. Potassium ferricyanide (<99%) and sodium hydroxide were obtained from Panreac (Barcelona, Spain), nickel chloride (<99%) from Êxodo Científica (Hortolândia, Brazil), and toluene (99.5%) from Synth (São Paulo, Brazil). Multi-walled carbon nanotubes (MWCNTs) (90%) were from Nanocyl^®^ NC7000^TM^ (Belgium) with an average diameter of 9.5 nm and an average length of 1.5 µm. Glucose and fructose were obtained from HenriFarma (São Paulo, Brazil), lysine was obtained from Farma Certa (Uberlandia, Brazil), urea from Dinamica^®^ (Indaiatuba, Brazil), sodium chloride, sodium phosphate dibasic, and sodium phosphate monobasic were obtained from Vetec (Duque de Caxias, Brazil) and calcium chloride, potassium chloride, and magnesium sulfate were obtained from Synth (Diadema, Brazil). The standard solutions of the interferents were prepared in 0.1 mol L^−1^ NaOH, which was the selected supporting electrolyte following the procedure reported elsewhere [37].

### 2.2. Apparatus

The electrochemical measurements (voltammetry and amperometry) were performed using a potentiostat/galvanostat, model PGSTAT 128N (Metrohm Autolab B. V., Utrecht, The Netherlands), connected to a notebook. The NOVA 1.11.0 software was used to register the measurements by the potentiostat. Electrochemical impedance spectroscopy (EIS) recordings were registered by a mini-potentiostat DropSens, model μStat-i 400s (Metrohm). As the counter electrode, a platinum wire was employed. As the reference electrode, a miniaturized silver-silver chloride electrode, constructed according to a previous publication, was employed [38]. As the working electrode, a glassy-carbon disk electrode (Ø = 1.5 mm) obtained from BASi Inc. (West Lafayette, IN, USA) was used (this electrode was used before and after surface modification).

Raman spectra were registered using a Horiba microscope, model LabRAM HR Evolution (Horiba, Kyoto, Japan), equipped with a 532 nm Argon laser with a working range of between 200 and 3500 cm^−1^. Different electrode regions were scanned by performing multiple spectra before and after the nickel oxy-hydroxide formation in the surface of the indium tin oxide (ITO) substrate. Infrared (IR) spectroscopic recordings were obtained using an FT-IR spectrophotometer, model Frontier Single Range (middle infrared (MIR)) from PerkinElmer (Waltham, MA, USA), which was connected to an attenuated total reflectance (ATR) accessory acquired from Pike Technologies (Madison, WI, USA). The spectra were obtained in the region between 4000 and 500 cm^−1^.

Amplified surface images were obtained using scanning electron microscopy (SEM) operated at 20 kV, acquired from Vega 3 LMU TESCAN, (Brno-Kohoutovice, Czech Republic). An energy-dispersed spectrometer (EDS), model INCA X-ACT (Stanford, Oxford, UK), coupled with the SEM equipment enabled the elemental composition.

### 2.3. Electrode Modification

**Figure 1:**
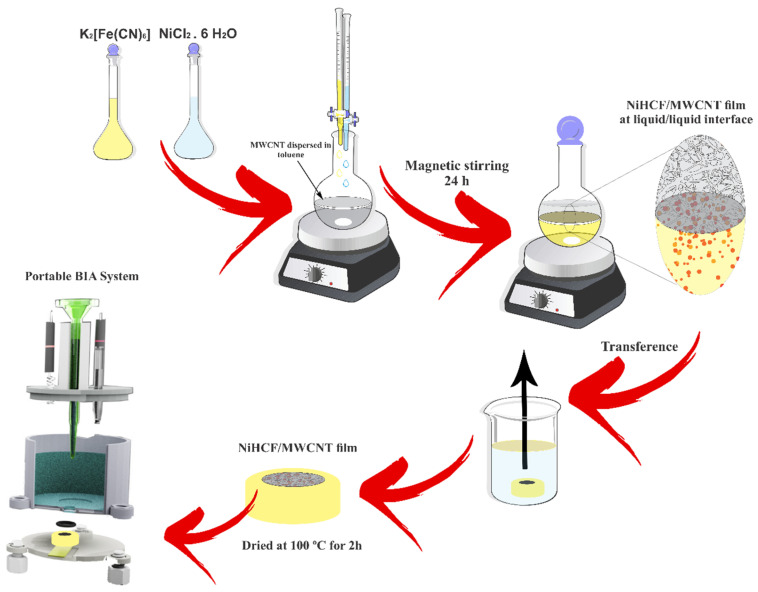
Schematic diagram of the deposition of NiHCF/MWCNT film on glassy carbon.

The liquid–liquid interfacial route is used for the synthesis of nickel hexacyanoferrate (NiHCF)/MWCNT film [35,36]. For the preparation of nanocomposite samples, specific amounts of MWCNT (0.15 mg) were added to 20 mL of toluene with subsequent dispersion using an ultrasonic probe (Cole-Parmer, model CV 18), 35% amplitude, and an application time of 15 min (sequential: ON for 50 s and OFF for 10 s). Next, 10 mL of both 0.01 mol L^−1^ NiCl_2_ and 0.1 mol L^−1^ K_3_[Fe(CN)_6_] were slowly added to this dispersion of MWCNT, forming a two-phase system. The two-phase system was maintained under strong magnetic agitation for 24 h. After 24 h, the magnetic stirring was stopped and a thin layer of transparent nanocomposite film was obtained at the water/toluene interface. The aqueous part of the biphasic system was washed three times with distilled water. The film formed at the interface was removed with a pipette and placed in a beaker containing water and glassy carbon substrates at the bottom. The self-assembled film was removed from the beaker with the suspension of the substrates, which were dried at a temperature of 100 °C for 2 h (Figure 1).

Then, the NiHCF/MWCNT-modified electrode was immersed in a 0.1 mol L^−1^ NaOH solution. The cyclic voltammetric parameters applied in this step were 50 mV s^−1^ as the scan rate and 0.0–0.7 V of potential range. The number of cycles was determined when both anodic and cathodic peak currents stopped increasing effectively, which was 50 cycles. After completion, the glassy carbon modified with Ni(OH)_2_/NiOOH/MWCNT was dried at room temperature.

### 2.4. Glucose Determination

Glucose sensing was carried out, employing the amperometric detection technique coupled with a batch injection analysis (BIA) cell (inner volume of 100 mL) as stated in the literature [39]. The cell was 3D-printed with ABS, which enabled the positioning of counter and reference electrodes through the top and the working electrode through the bottom. The injector (Eppendorf^®^ electronic pipette) was connected through the top. Injection volume and rates were optimized. The experiments using the BIA system with amperometric detection were performed with the solution (supporting electrolyte) inside the cell under stirring using a commercial magnetic stirrer (Topolino IKA^®^). In this condition, after injection steps, the baseline current was re-established more quickly (narrower BIA peaks).

### 2.5. Artificial Blood Plasma Analysis

The applicability of the nickel oxy-hydroxy/MWCNT for the determination of glucose was evaluated using an analysis of artificial blood plasma samples (for 1 L: NaCl 6.8 g, CaCl_2_ 0.2 g, KCl 0.4 g, MgSO_4_ 0.1 g, NaHCO_3_ 2.2 g, Na_2_HPO_4_ 0.126 g, NaH_2_PO_4_ 0.026 g) [40]. For the amperometric measurements, the working solutions were prepared and diluted (50-fold) in the NaOH electrolyte solution (0.1 mol L^−1^) by adding glucose standard to the artificial blood plasma samples.

## 3. Results and Discussion

### 3.1. Synthesis of Nickel Oxy-Hydroxide/MWCNT Nanocomposite Film

**Figure 2:**
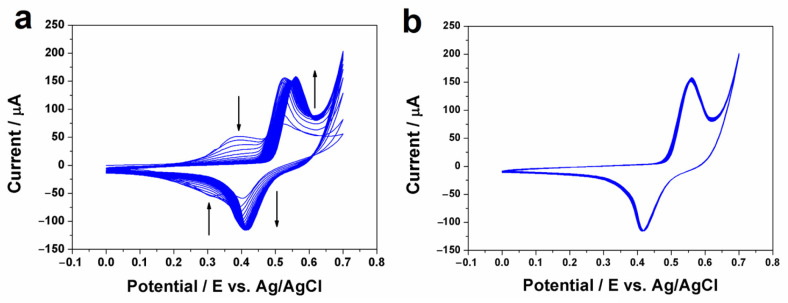
(**a**) Voltammetric behavior corresponding to 50 cycles (0.0 to +0.7 V) of the NiHCF/MWCNT film deposited on GCE, continuously recorded in 0.1 mol L^−1^ NaOH at 50 mV s^1^. (**b**) Voltammetric behavior corresponding to the NiOOH/Ni(OH)_2_ film formed after 25 cycles.

A glassy carbon electrode, modified with a nickel oxy-hydroxide/MWCNT nanocomposite film, was prepared in two steps. Firstly, the NiHCF/MWCNT nanocomposite was prepared using the interfacial method [35,36]. Then, the NiHCF/MWCNT nanocomposite was converted into nickel oxy-hydroxide/MWCNT by cyclic voltammetry using the parameters mentioned in the previous experimental section. Figure 2a shows the voltametric profiles of the nickel oxy-hydroxide formation, with which it is possible to observe the presence of two electrochemical processes. In the potential region of +0.31/0.39 V, the redox process can be attributed to the reduction and oxidation of iron sites present in the NiHCF structure. The alkaline medium favors the decomposition of NiHCF and, consequently, the formation of nickel oxy-hydroxide, which is characterized by the appearance of two redox peaks related to the NiO(OH)/Ni(OH)_2_ (+0.46/0.55 V). This electrochemical process can be expressed according to the following equation:

Ni(OH)_2_ + OH^−^ ⇄ NiO(OH) + H_2_O + e^−^ (1)

Additionally, both cathodic and anodic peaks gradually increased during successive scans. The oxidation process was described by Ballotin and coauthors [33]; they showed that the electrochemical stability of NiHCF in an alkaline solution is poor, promoting the formation of nickel oxide on the electrode surface by successive scans.

As the range of successive scans increase, a higher current density is observed and sharp peaks appear (+0.46/0.55 V). After five cycles, the complete conversion of the NiHCF into the NiOOH/Ni(OH)_2_ is observed, characterized by the disappearance of redox peaks +0.31/0.39 V. Figure 2b shows the high stability of NiOOH/Ni(OH)_2_ between the 25th and 50th cycles, which can be explained with the π-π interaction between the inorganic material and MWCNTs, where the carbon material acts as an electron donor and the nickel oxy-hydroxy as the acceptor [35,41].

### 3.2. Morphology and Structural Properties of Nickel Oxy-Hydroxide/MWCNT

**Figure 3:**
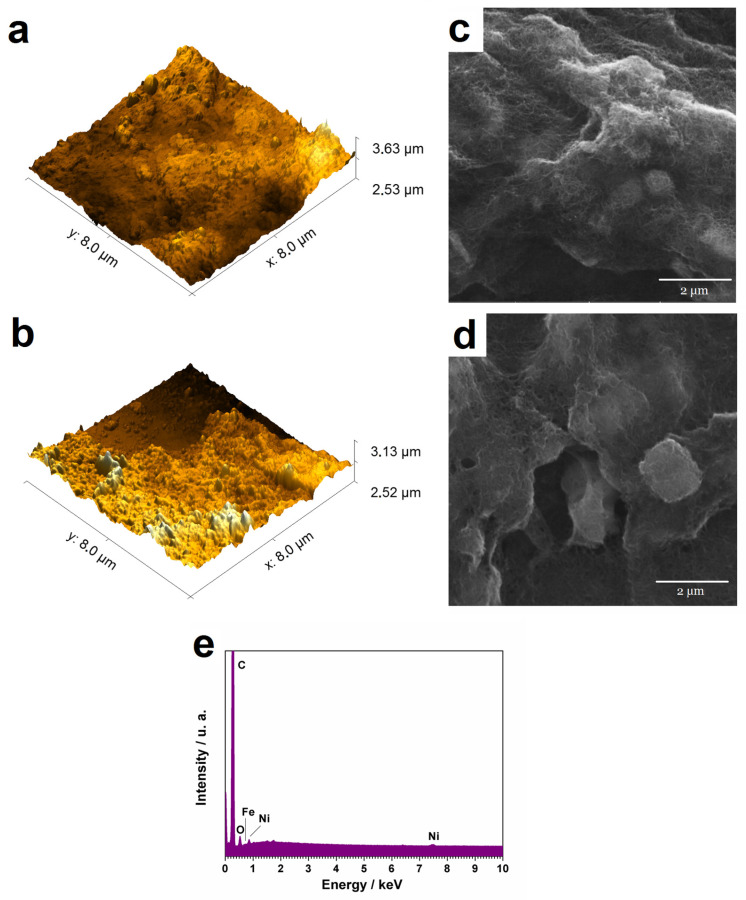
AFM and SEM images (**a**,**b**) of nickel hexacyanoferrate/MWCNTs film and (**c**,**d**) of nickel oxyhydroxide/MWCNTs electrode. (**e**) EDS pattern of nickel oxyhydroxide/MWCNT.

The morphology and microstructure of the nickel hexacyanoferrate/MWCNT and nickel oxy-hydroxide/MWCNT films are shown in the AFM and SEM images (Figure 3). The SEM images reveal that both nanocomposite films are formed by nanoparticles with irregular cubic morphology. From the AFM images, the rugosity was calculated for both surfaces and the values are 14.35 and 10.52 nm for NiHCF/MWCNT and nickel oxy-hydroxy/MWCNT, respectively. Both electrodes present the same characteristic homogeneity and rough surface, which may improve the electrochemical response as will be further discussed. In addition, the contact between nickel oxy-hydroxide particles and the MWCNTs can also be observed. The EDS analysis (Figure 3d) confirms the presence of Ni and O, associated with the nickel oxy-hydroxide particles, and Fe related to the presence of NiHCF. Moreover, the signal of C assigned to the MWCNTs is also observed. These data corroborate with the materials proposed during interfacial and electrochemical synthesis.

**Figure 4:**
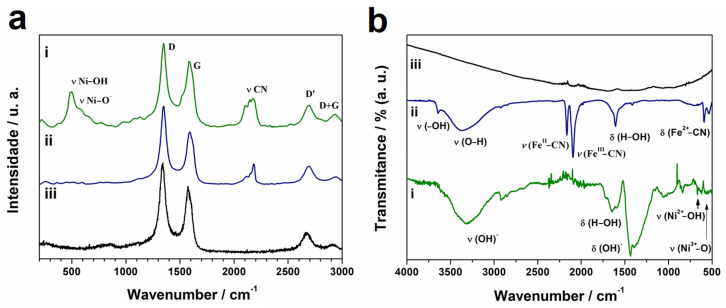
(**a**) Raman spectra and (**b**) infrared spectra of (i) nickel oxyhydroxide/MWCNT; (ii) NiHCF/MWCNT and (iii) MWCNT.

Raman spectroscopy was adopted to illustrate the chemical structure and confirm the nickel oxy-hydroxide formation on the modified electrode. Raman spectra were collected from the surface of the electrode before and after cycling in the NaOH solution, that is, before and after the formation of nickel oxy-hydroxide (Figure 4a). All spectra present the D (1348 cm^−1^), G (1580 cm^−1^), 2D (2691 cm^−1^), and D + G (2936 cm^−1^) bands characteristics of MWCNT (Figure 4a(iii)). The D band is generally known for its disorder-induced mode, which is derived from the presence of defects/borders in the MWCNTs, whereas the G band is associated with the overbending of sp^2^ carbon–carbon bonds of longitudinal optical branches of MWCNT. The 2D band is attributed to interplanar stacking order. Before the electrosynthesis of nickel oxy-hydroxide (Figure 4b(i)), the spectrum is characteristic of the formation of an NiHCF/MWCNT nanocomposite, with the presence of a band at 2183 cm^−1^ related to C≡N stretching. After the formation of nickel oxy-hydroxide/MWCNT, the spectrum (Figure 4a(ii)) shows new bands characteristic of nickel oxy-hydroxide, at 494 cm^−1^ and 588 cm^−1^, assigned to the Ni-OH and Ni-O stretching vibrations, respectively [42,43].

The IR absorption spectra of nickel oxy-hydroxy/MWCNT (i), NiHCF/MWCNT (ii), and MWCNT (iii) are shown in Figure 4b. The sharp peak at 3650 cm^−1^ refers to the hydroxyl groups’ (-OH) stretching vibration mode, the absorption band at 3400–3050 cm^−1^ in the nickel oxy-hydroxy/MWCNT and NiHCF/MWCNT samples due to O-H stretch, and the band at 1650 cm^−1^ (H-O-H bending mode) can be assigned to the absorption of H_2_O in the film. The band at 2100 and 2170 cm^−1^ related to C≡N stretching in the NiHCF/MWCNT sample disappears after the formation of the nickel oxy-hydroxy/MWCNT composite. The bands around 671 and 563 cm^−1^ are described as Ni^2+^-O and Ni^3+^-O bending vibration from the NiOOH [44]. The spectrum of GCE modified with MWCNT did not present relevant peaks in the region of 500–4000 cm^−1^ (Figure 4b(iii)) due to the absence of oxygenated functional groups and the low presence of defects in the MWCNT structure.

### 3.3. Electrochemical Measurements

#### 3.3.1. Electrochemical Characteristics of the Modified Electrodes for Glucose Detection

**Figure 5:**
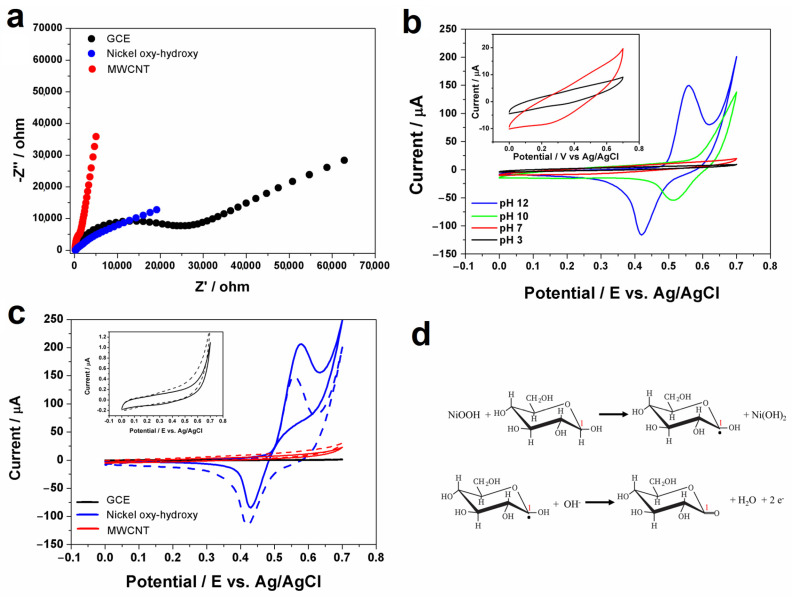
(**a**) The EIS of GCE electrode, MWCNT, and nickel oxy-hydroxy/MWCNT in 5 mmol L^−1^ of K_3_Fe(CN)_6_/K_4_Fe(CN)_6_ and 0.1 mol L^−1^ KCl solution in the frequency range swept 10^5^–0.1 Hz at 0.28 V. (**b**) Cyclic voltammograms of NiOOH/Ni(OH)_2_ film in pH: (−) 3.0; (−) 7.0; (−) 10.0; (−) 12.0 (ν = 50 mV s^−1^). (**c**) CV measurements recorded at GCE, MWCNT, and nickel oxy-hydroxy/MWCNT without (dashed lines) and with (full lines) the presence of glucose 0.1 mM glucose. supporting electrolyte: 0.1 M NaOH solution; scan rate: 50 mV s^−1^; step potential: 5 mV. (**d**) Illustration of schematic representation of glucose oxidation to glucolactone mechanism on nickel oxy-hydroxy/MWCNT electrode.

The electrochemical impedance spectroscopy (EIS) experiment was performed in 5 mmol L^−1^ of K_3_Fe(CN)_6_/K_4_Fe(CN)_6_ in a 0.1 mol L^−1^ KCl solution. As shown in Figure 5a, the Nyquist diagrams of GCE electrode, MWCNT, and nickel oxy-hydroxy/MWCNT consist of a semi-circular profile at higher frequencies and a linear shape at lower frequencies, corresponding to the electron transfer limited process and diffusion-controlled process, respectively. MWCNTs remarkably reduce the impedance of GCE, while nickel oxy-hydroxy/MWCNT presented a considerable semi-circle, which indicates that modified surfaces may contribute to an improved electron transfer. The effect of pH on the electrochemical behavior of NiOOH/Ni(OH)_2_ was investigated by cyclic voltammetry at a pH range of 3.0 to 12.0 (Figure 5b). With the enhancement of pH value, it is observed in voltammetry cycles that the electrochemical processes are strongly influenced by the OH^−^ concentration of the medium. Additionally, with the increasing concentration of OH^−^ ions in the medium, the potential shifted towards more negative values and the peak current increased, indicating the influence of the protonation on the electrochemical process.

Figure 5c shows cyclic voltammograms recorded at unmodified GCE (black line), GCE modified with MWCNT (red line), and GCE modified with nickel oxy-hydroxy/MWCNT (blue line) in the absence (dashed lines) and presence (full lines) of 1 mmol L^−1^ glucose. After the addition of glucose, the GCE modified with nickel oxy-hydroxy/MWCNT film presents an improvement in the anodic current and a decrease in the corresponding cathodic current, which can be indicative of the electrocatalytic activity of nickel oxy-hydroxy/MWCNT for glucose oxidation. Glucose is not electrochemically active on carbon-based materials, as can be seen in Figure 5 (red and black lines). A widely used strategy described in the literature for the selective and indirect electrochemical detection of glucose is the use of electrodes modified with glucose oxidase. However, enzyme-based sensors have some disadvantages, such as the instability of the enzyme (requirement of temperature, pH, and humidity control) and short electrode lifetime, which can limit the applicability of this type of sensor [45,46].

In this sense, transition metal-based materials have been a promising alternative for glucose determination, including platinum, copper, gold, and nickel [45]. The electrocatalytic process is usually performed by the adsorption of the analyte on the electrode surface, which involves the d-electrons presented in the metals of the substrate [45,47].

According to previous studies, the exposure of nickel-based materials to an alkaline solution results in the transformation of portion Ni-based materials to nickel hydroxide (Ni(OH)_2_), and the Ni(OH)_2_ is the main contributor to the electrocatalytic oxidation of glucose to glucolactone, according to the following reaction [18]:

NiOOH + glucose → Ni(OH)_2_ + gluconolactone (2)

This catalytic oxidation process is shown in Figure 5d. In an alkaline solution, oxygen atoms on NiO(OH) easily interact with hydroxyl groups of glucose, promoting the hydrogenation and conversion of glucose to glucolactone. This process depends on the concentration of OH- and a high pH value is required [48]. So, the increase in the anodic peak current in cyclic voltammograms observed in the presence of 1 mmol L^−1^ glucose (Figure 5c) is related to the mechanism reaction described above. Moreover, a reduction in the cathodic peak can be attributed to the consumption of NiOOH by glucose as previously described in the literature [45].

Furthermore, the catalytic effect of NiOOH can also be improved by the excellent conductivity and high surface area of MWCNTs. A previous work stated that the catalytic active sites for glucose oxidation—NiOOH—are electrically connected by MWCNTs in such a way that the NiOOH particles are anchored on MWCNT particles [41]. The combination of the high conductivity of MWCNTs and the electrochemical activity of NiOOH favors the low oxidation potential for glucose sensing [32].

#### 3.3.2. Amperometric Detection

**Figure 6:**
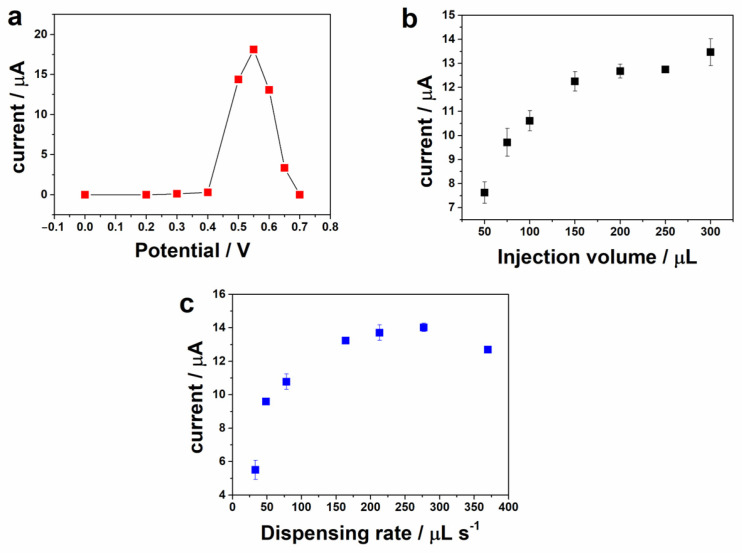
(**a**) Plot of current (*n* = 3) as a function of potential under hydrodynamic conditions determined by the BIA system. Effect of (**b**) injection volume and (**c**) dispensing rate on the current response for glucose. Working potential: +0.55 V; supporting electrolyte: 0.1 mol L^−1^ NaOH; concentration of glucose: 100 μmol L^−1^; dispensing rate: 154 μL s^−1^ in (**a**); injected volume: 200 μL in (**b**).

To demonstrate the possibility of glucose determination, the glassy carbon electrode modified with nickel oxy-hydroxy/MWCNT was coupled to a BIA system with amperometric detection (practical and portable system). Some experimental parameters of the BIA system (applied potential, injection volume, dispensing rate, and use of stirring device) were evaluated to achieve the best sensitivity, fast response time, and good reproducibility in the analysis. Figure 6a was constructed using amperometric current obtained for triplicate injections of 100 μmol L^−1^ glucose as a function of applied potentials (+0.3 V to +0.7 V vs. Ag/AgCl). The highest current was obtained at +0.55 V since NiOOH formation is necessary for glucose oxidation (as can be observed in the cyclic voltammograms in Figure 4). Thus, based on this experiment, the potential of +0.55 V (vs. Ag/AgCl) was selected for further BIA experiments. The effect of BIA parameters, such as injection volume and dispensing rate, were also evaluated and the obtained results are shown in Figure 6b,c. As expected, the detected current increased with the increase in both volume and rate of injection. The negligible gain was verified in extreme conditions (>200 µL and >213 µL s^−1^). Thus, these values were selected for the next experiments.

**Figure 7:**
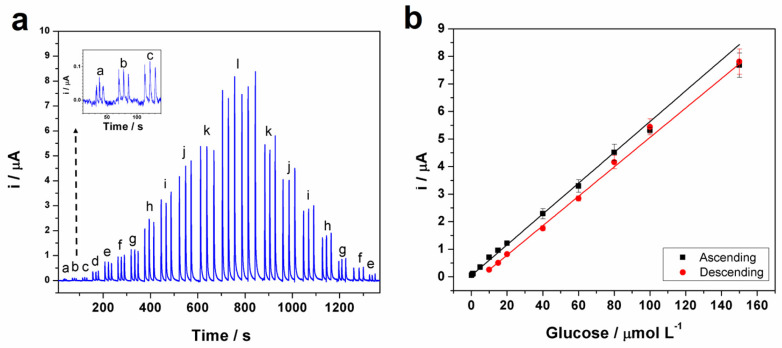
(**a**) BIA amperogram recorded at +0.55 V for triplicate injections of solutions containing increasing and decreasing glucose concentrations: (a) 0.1, (b) 0.5, (c) 1, (d) 5, (e) 10, (f) 15, (g) 20, (h) 40, (i) 60, (j) 80, (k) 100, (l) 150 µmol L^−1^. (**b**) Calibration curves obtained from data presented in (**a**). Working electrode: GCE modified with the nickel oxyhydroxide modified; supporting electrolyte: 0.1 mol L^−1^ NaOH; dispensing rate: 213 µL s^−1^; injection volume: 200 µL.

Figure 7a,b displays the amperogram and respective calibration curves (ascending and descending order) obtained with the injection of solutions containing increasing concentrations of glucose. The limit of detection (LOD) was calculated based on the IUPAC definition (3σ/s), where *s* is the slope of the calibration curve and σ is the standard deviation of baseline noise. The sensitivity was found to be 0.0561 μA μmol L^−1^ and a linear range from 0.1 to 150 μmol L^−1^ was obtained with a good limit of detection (0.030 μmol L^−1^). Therefore, the sensor exhibits a fast response (150 injections h^−1^) and a sensitive catalytic performance, which may be due to the high conductivity of MWCNT and the increased active surface area of the electrode. Additionally, the minimal difference in the sensitivity for ascending (0.0561 µA µmol L^−1^) and descending (0.0531 µA µmol L^−1^) calibration curves indicates no memory effects on the electrode surface (no electrode fouling). This result also shows the high stability of the sensor for continuous monitoring using the BIA system.

**Figure 8:**
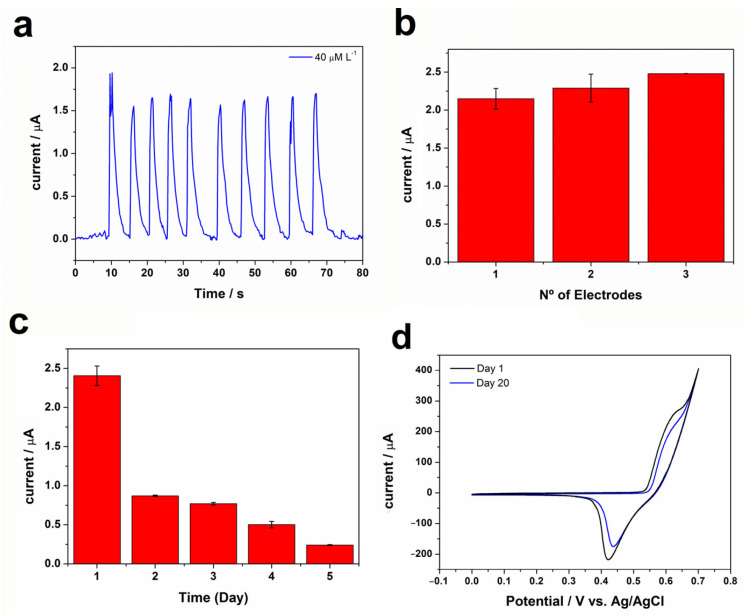
(**a**) Amperogram obtained for successive injections (*n* = 10) of glucose in concentrations of 40 μmol L^−1^. (**b**) Stability test for 40 μmol L^−1^ of glucose for 5 days and (**c**) current response at 3 different nickel oxy-hydroxy electrodes for 40 μmol L^−1^ of glucose. (**d**) Cyclic voltammogram stability of nickel oxyhydroxy/MWCNT.

The repeatability of the proposed BIA method was examined with ten successive measurements of 40 μmol L^−1^ of glucose using the nickel oxy-hydroxy/MWCNT electrode (Figure 8a). In this study, a relative standard deviation (RSD) of 6.7 % (*n* = 10) was obtained, which shows that the electrode response is highly stable. Three different nickel oxy-hydroxy/MWCNT electrodes were prepared using the same method and were used to detect 40 μmol L^−1^ glucose (Figure 8b). A low RSD value (7.2%; *n* = 3) was obtained, indicating good inter-electrode reproducibility. The storage stability of the nickel oxy-hydroxy/MWCNT electrode was investigated by adding 40 μmol L^−1^ of the analyte in each system. The nickel oxyhydroxy/MWWCNT modified electrode lost 64% and 90% of the current response after 1 and 5 days, respectively (Figure 8c), after successive uses. This result indicates the instability of nickel oxyhydroxy/MWCNT on the electrode surface over those days. However, the stability of the oxyhydroxy/MWCNT electrode after the synthesis process was investigated and no decrease in the current intensity of the anode and cathode peaks was observed after 20 days (Figure 8d), which indicates a high accuracy and high shelf-life of the electrode. This result indicates the instability of nickel oxyhydroxy/MWCNT on the electrode surface over the days; however, the ease of manufacture, low cost, and the possibility of deposition on different conductive substrates makes the development of disposable electrochemical sensors promising.

#### 3.3.3. Analytical Application of the Nickel Oxy-Hydroxy/MWCNT Electrode: Glucose Sensing in Artificial Plasma

**Figure 9:**
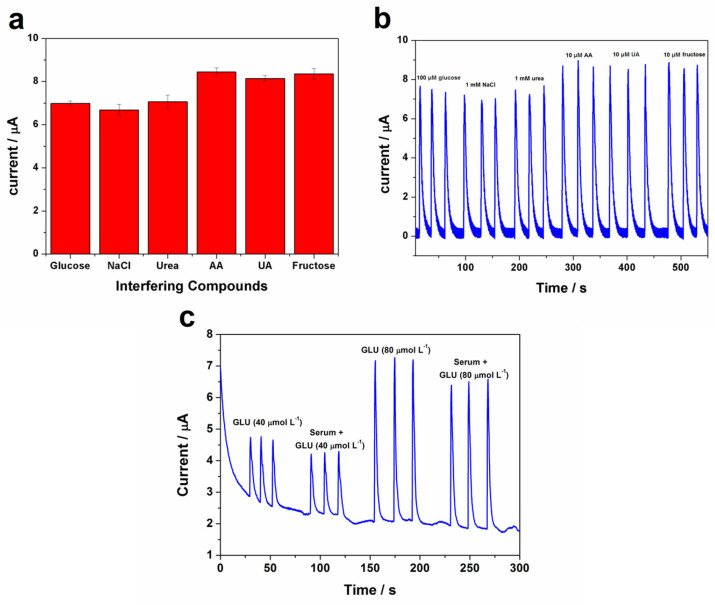
(**a**,**b**) Amperometric response for 100 μmol L^−1^ of glucose and 0.1 μmol L^−1^ of interfering molecules (*n* = 3) at nickel oxy-hydroxy/MWCNT electrode. (**c**) NaOH electrolyte solution spiked with 40 and 80 µmol L^−1^ glucose in the absence (GLU) and in the presence (Serum + GLU) of artificial blood plasma sample (50-fold diluted).

The glucose level in human blood is in the millimole range and, usually, the amount of glucose is at least 30 times higher than the content of interferents compounds. Thus, the ratio of glucose concentration as a function of interfering species was kept at 10:1 [49]. The concentration of 10 μmol L^−1^ was kept for interferents (ascorbic acid, uric acid, and fructose) mixed with 100 μmol L^−1^ glucose, and the results are presented in Figure 9a,b. It is observed that urea, AA, UA, and fructose have a slight increase in the current response of glucose; however, the different intensity of just around 10% does not interfere in the current considering the levels of the interfering compounds in normal physiological conditions [50,51]. Moreover, chloride ions may cause blocking on the metallic oxide-based sensor and, as a consequence, decrease its activity; however, the nickel oxy-hydroxy/MWCNT electrode prepared in this work shows a good anti-interference ability in terms of NaCl, indicating that the modified electrode is immune to the poisoning by Cl^−^ [24].

As noticed in preliminary studies, glucose sensors often encounter challenges in detecting glucose accurately in complex biological samples, such as blood and urine, which contain other interfering compounds that can affect sensor performance. In this case, the proposed nickel oxy-hydroxide/MWCNT is a non-enzymatic sensor for the determination of glucose in artificial blood plasma. As shown in Figure 9c, the artificial blood plasma sample (50-fold diluted) spiked with 40 and 80 µmol L^−1^ glucose displayed a similar current compared with glucose in the NaOH electrolyte solution. Using this protocol, recovery values of 98 and 89% were achieved for 40 and 80 µmol L^−1^ glucose, respectively, indicating that the developed approach has high accuracy and selectivity in measuring glucose concentrations in artificial blood plasma, showing the potentiality of the electrode in real sample analysis, and a reliable and affordable alternative platform for glucose sensing, requiring only simple sample preparation (dilution in the electrolyte).

The performance of the nickel oxy-hydroxide/MWCNT is comparable with other recent non-enzymatic glucose sensors based on carbon structures and nickel-based composite materials (Table 1). It can be seen that the nickel oxy-hydroxy/MWCNT sensor has a lower limit of detection compared with the other sensors (similar values to Pd-Ni@f-MWCNT and NiO/FTO), with comparable or higher sensitivity, so the sensor proposed in this work exhibits prominent electro-catalytic ability towards glucose and may be applicable as a low-cost and easily manufactured non-enzyme glucose sensor.

Another important feature is that the coupling of the proposed sensor with the BIA provided fast analysis in such a way that 150 injections of solutions (standard or sample) were injected per 1 h. This work presents a successful example of BIA combined with a modified electrode for glucose sensing using a completely portable system [62]. It is important to highlight that BIA-AMP cells cost around USD 10.00 [63] and the electrode modification can be easily fabricated, which allows for the economical and miniaturized construction of lab-on-chip devices.

## 4. Conclusions

In summary, a nickel oxy-hydroxy/MWCNT nanocomposite-modified electrode was successfully synthesized using cyclic voltammetry for the development of a non-enzymatic sensor. The interaction between nickel oxy-hydroxy and the MWCNTs provided a film that is stable over the electrode surface, with high surface area and excellent conductivity. Glucose sensing benefitted from the electrocatalytic activity of the nanocomposite formed within the film. The BIA system with amperometric detection showed an excellent analytical performance (LOD = 0.03 μM; sensitivity = 0.0561 μA L μM^−1^; analytical frequency = 150 injections h^−1^) for the determination of glucose, with the nickel oxy-hydroxy exhibiting a low detection limit (based on a signal-to-noise ratio of 3) and high sensitivity (0.0561 μA L μM^−1^). The nickel oxyhydroxy/MWCNT is unstable on the electrode surface over the days; however, the ease of manufacture, low cost, and the possibility of deposition on different conductive substrates make the development of disposable electrochemical sensors promising, and the practicality of the BIA system makes this tool a promising candidate for routine glucose sensing.

## Figures and Tables

**Table 1:** Performance comparison of nickel oxyhydroxide/MWCNT with other non-enzymatic nickel-based glucose sensors.

Electrode Material	Technique	Sensitivity	Potential/V	Linear Range/mmol L^−1^	LOD/µmol L^−1^	Ref
NiO/C porous	AMP	2918.2 (µA mmol^−1^ cm^−2^)	0.55	0.005–4.1	0.92	[52]
Pd-Ni@f-MWCNT	AMP	71 (µA mmol^−1^ cm^−2^)	0.55	0.01–1.4	0.03	[53]
NiNPs/ERGO	AMP	185.2 (µA mmol^−1^ cm^−2^)	0.55	0.0005–0.244	0.04	[54]
nickel oxyhydroxide/MWCNT	CV	32 (µA mmol^−1^ cm^−2^)	0.52	0.4–5.0	230	[55]
nickel oxyhydroxide/MWCNT	CV	0.00013 (µA L µmol^−1^)	0.55	0.25–5.6	190	[56]
NiO/MWCNT	CV	436.3 (µA mmol^−1^ cm^−2^)	-	0.2–12	160	[57]
NiO/FTO	AMP	17,485.41 (µA mmol^−1^ cm^−2^)	0.5	0.001–0.27	0.033	[58]
Ni/NiO/rGO-Screen Printed electrode	AMP	1997 (µA mmol^−1^ cm^−2^)	+0.55	0.0299–6.44	1.8	[59]
NiO/Cu-TCPP	AMP	4600 (µA mmol^−1^ cm^−2^)	+0.55	0.00285–0.2885	0.95	[60]
N-doped-Carbon nanofiber NiOx	CV	-	-	0.5–10	-	[61]
nickel oxyhydroxide/MWCNT	BIA-AMP	0.0561 (µA L µmol^−1^)	0.55	0.001–0.150	0.03	This work

## Data Availability

Data sharing is not applicable to this article.
